# The influence of gastronomic identity factors on food tourism development in the Republic of Serbia and Bosnia and Herzegovina

**DOI:** 10.3389/fnut.2023.1335943

**Published:** 2024-01-08

**Authors:** Bojana Kalenjuk Pivarski, Maja Paunić, Nemanja Šarenac, Stefan Šmugović, Predrag Mlinarević, Velibor Ivanović, Jelena Marjanović, Stevan Pavlović, Dragana Tekić, Bojan Ðerčan, Dragan Tešanović, Snježana Gagić Jaraković

**Affiliations:** ^1^Department of Geography, Tourism and Hotel Management, Faculty of Sciences, University of Novi Sad, Novi Sad, Serbia; ^2^Faculty of Economics Pale, University of East Sarajevo, Pale, Bosnia and Herzegovina; ^3^Department of Agricultural Economics and Rural Sociology, Faculty of Agriculture, University of Novi Sad, Novi Sad, Serbia; ^4^International Center for Professional Studies, Novi Sad, Serbia

**Keywords:** food, gastronomy, tourism, gastronomic tourism, catering, Serbia, BIH

## Abstract

**Background and aims:**

The gastronomic identity of an area is the key factor in tourism development, attracting numerous tourists and generating significant income. Numerous economic actors participate in its use and proper placement, and their perception of the gastronomic potential significantly affects its distribution and use in tourism. The main aim of this study is to investigate the factors of gastronomic identity that influence the development of tourism, observed at two tourist destinations in Southeast Europe [the Republic of Serbia (RS) the city of Novi Sad with Fruška Gora Mountain, *n* = 305 and Bosnia and Herzegovina (BIH) the city of Sarajevo with Jahorina Mountain, *n* = 301].

**Methods:**

In order to define the factors that are relevant to food tourism development, a custom-made GastroIdentity scale was created. A survey was conducted among employees in the hospitality and tourism industry as well as employees in educational institutions in the field of hospitality and tourism.

**Results:**

The research results show that employees from the RS area acknowledge the importance of organizing gastronomic events where local products are presented and that they understand that dishes and beverages with unique and recognizable tastes can characterize their area. Employees from the BIH area pointed out that the nutritional quality of their local agricultural and gastronomic products represents an advantage when compared to mass-produced ones and that the local gastronomic culture and tradition are authentic representatives of the culture of the region.

**Conclusion:**

The GastroIdentity scale proved to be dependable, highlighting gastronomic culture and tradition as extremely crucial factors in tourism, using the input provided by the employees from the investigated areas. Noteworthy results were also recorded regarding the need for incentives for food tourism development in the investigated regions.

## 1 Introduction

The gastronomic identity of any location is extremely important for tourism development and attracting tourists (1). A special focus is put on the increasingly popular food tourism, which attracts tourists with the spending power, ready to pay for the high quality and unique gastronomic experience (2). Activities that include this selective form of tourism comprise visits to various food producers, catering establishments, events, and culinary schools, where food is the primary motive of the tourist movement (3, 4), the variations of which are often called gastronomic tourism, tasting tourism, gourmet tourism, etc. (5).

When discussing the significance of implementing gastronomy and improving hospitality and tourism, it is greatly important to emphasize all the economic and social benefits, accomplished by the improvement and increased tourist attractiveness of an area. This potential has been recognized in various destinations that were analyzed in terms of the most important elements contributing to this, such as gastronomic heritage, credibility, and originality in tourism (6, 7). Employees in the hospitality industry are indispensable in the perception of the relevant factors that can be beneficial for food tourism development. Nevertheless, there is a lack of research on this topic from the employees' point of view even though they are the key creators of the offer primarily aimed at tourists and the local people (8, 9). Therefore, this is the principal reason for conducting this research.

This study is aimed at defining the gastronomic identity factors that influence the development of food tourism in the selected tourist areas in the Republic of Serbia and Bosnia and Herzegovina. The significance of conducting this research is multi-faceted. Both regions belong to the developed areas where substantial agricultural activity is present in the vicinity of 100 km as well as consumption in restaurants (10). At the same time, both areas are multicultural, which is extremely valuable for gastronomic identity assessment by all tourism stakeholders (11). The main aim of the study is to identify the factors that stand out in the recognition of the gastronomic identity of the studied areas, from the viewpoint of people working in the catering and tourism industry, and educational institutions, which are considered to be the key creators of this identity. The study will provide answers to the research questions based on the concepts presented in the literature review in the next section, which will also contribute to taking adequate action on the tourism market of the Republic of Serbia and also Bosnia and Herzegovina.

## 2 Literature review

### 2.1 Development of food tourism in correlation to gastronomic identity as a reflection of gastronomic culture and tradition

Food tourism represents an important form of tourism that allows tourists to experience a specific destination in a specific way, tasting and enjoying different types of food, agricultural, and gastronomic products (12–14). Research has shown that nurturing and using authentic agricultural products and other local ingredients in the preparation of authentic dishes not only attracts visitors to the destination but also significantly extends their stay (15, 16). The potential that food tourism has for the development of a certain destination is reflected in the growing interest in local gastronomy among foreign visitors as well as the desire to try and taste local and perhaps lesser-known dishes and beverages (17, 18). This form of tourism includes various activities such as gastronomic routes, tourist events, culinary schools, and tastings, which can accelerate and improve the transformation and use of traditional food from rural regions, turning them into an attractive offer on the national market (19, 20).

In addition to the aforementioned gastronomic routes, food tourism provides tourists with the opportunity for actively participating in the preparation of local food, which significantly contributes to the feeling of satisfaction among tourists and thus to the development of the gastronomic identity of a destination (21). It is interesting to mention that Chang et al. (22) stated that the experience of personal participation in the preparation of food is the most important of the twenty-two criteria for the development of food tourism as one of the selective forms of tourism.

The satisfaction of tourists who are motivated to explore the gastronomy of a destination is closely related to the relationship that gastronomy has with the cultural heritage of the local community (23). Local gastronomy is recognized as a component of regional cultural heritage, reflecting the historical narrative, customs, and traditions of a community or a geographic area and serving as a means to shape the distinctive identity and cultural profile (24–26). This confirms that the development of the gastronomic brand of a destination is not possible without establishing and developing the gastronomic heritage and culinary tradition (27).

Popular gastronomic products can grow and become a brand if the entire gastronomy and culinary practices are valued as an important segment of cultural heritage and tourism (28). Dietary choices represent a series of complex decisions that not only have a significant impact on the environment but also on the development of gastronomy, making food culture a significant factor in establishing and defining gastronomic identity in tourism (29).

Having considered the abovementioned concepts, the first research question (Q1) would be as follows: Which elements of gastronomic identity are the most important for food tourism development in the selected tourist destinations?

### 2.2 Important factors that create gastronomic identity and contribute to food tourism development—tourism events and vendors

Gastronomic identity consists of numerous factors that represent strategic resources of differentiation and competitive advantages of a destination (30). There are numerous factors that influence the creation of a gastronomic identity, which is important for tourism development. Gastronomic identity is shaped by elements such as climate, geographic positioning, economic prosperity, and the extent of influence of other cultures (31). Establishing the gastronomic identity of a destination contributes to the development of a sense of loyalty, which can also be shown in the examples of gastronomic events, which are of great importance and value for visitors (32), as well as factors such as gastronomic culture, food quality, catering facilities, namely, different types of gastronomic activities (8).

Food and beverage events are different forms of fairs, festivals, exhibitions, and cultural and industry events that are held regularly or only once. A significant increase in the number of gastronomic events has contributed to the promotion and development of many tourist and economic destinations (33). In today's world, with the aim of preserving the cultural identity of a nation, more than 580 gastronomic events are organized in 30 countries, whose activities are aimed at preserving, building, and nurturing the gastronomic heritage of a nation. Gastronomic events are a unique link between the consumption of food, beverages, travel, and events. They involve gathering a large number of people in one place with the aim of trying traditional food and beverage products and observing or participating in the preparation of dishes. These events have become greatly important for the development of a destination and tourism (34, 35).

According to Vićentijević (36), events play a significant role in the improvement of the tourist offer in Serbia, especially when, in addition to the original motive of the visit, they additionally motivate the visitor to make a decision in order to get to know the gastronomic offer. Food events not only bring benefits to food producers and local businesses by attracting local residents and tourists but also have an economic impact on the region and increase the awareness of the area as a destination for food tourism and the presentation of local gastronomy and identity (36, 37).

Based on the aforementioned insights and methodology, the second research question is asked to establish the situation in the investigated area (Q_2_): Is the created scale for measuring the factors justified, and which data are obtained?

### 2.3 Gastronomic identity in the context of local food and its characteristics—quality

Gastronomic identity plays an important role in destination marketing, especially where food quality is considered exceptional. The issue of quality is also an important topic because this directly affects tourists' purchase intentions. Therefore, high-quality food has a great potential for expansion as a primary motive for visiting a tourism destination, especially because it attracts very selective groups of tourists with extra money to spend on high-value, unique, and high-quality products (38, 39).

Rahman et al. (40) stated that tourists who perceive local food as high quality are more determined to purchase it. For this reason, it is important to emphasize that the different characteristics of local food represent an important factor in the preferences of different tourists. Research has shown that some tourists emphasize freshness and health benefits as a significant advantage of local over commercial products (41), while others emphasize the quality of food (42). Similarly, research conducted by Kovács et al. (43) showed that tourists associated local food with characteristics such as freshness, health, care for the environment, high nutritional value, and many others. Food quality attributes depend on the product type and personal preferences of the consumer. The characteristics that are associated with quality by the consumers can change over time, but the consumers themselves can change their minds about the quality and remain dissatisfied with the final product that does not meet their expectations (44).

In fact, some studies have shown that the satisfaction of food tourism participants is mainly influenced by four factors: food quality, service quality, physical environment, and price. Food quality is the most important determinant of satisfaction and loyalty to a specific product and offer, while service quality is an important element related to customer satisfaction. Improving the quality of food and final products increases consumer satisfaction, strengthens loyalty, and affects the sustainability and stability of the overall experience for customers and tourists (45, 46).

The personal contact of the customer with the producer is the first advantage of direct sales of domestic products. Due to the growth of the trend of preparing meals using local traditional products, the need to purchase these products has also increased. On the basis of the conducted studies, clearly defined disadvantages as well as advantages of using local products and preparing and offering meals at the host's business establishment were established. In addition to the many advantages of using domestic traditional products, support for the local economy, procurement of smaller quantities of products, freshness, and food safety, the knowledge of products and production practices are highlighted (47, 48). Labeling and certification systems are also extremely useful in distinguishing the quality of traditional products and representatives of the gastronomic identity of a tourist destination (25, 49, 50).

The positive synergy between the tourism and hospitality sectors fosters the sustainable and healthy growth of food tourism and catering, especially within an environment equipped with strong support and monitoring systems (51, 52). Incentives and different types of assistance are important in ensuring the quality of gastronomic products and services, primarily for small producers and providers of unique and authentic food products (53). Financial incentives play an important role in adequately marketing gastronomic products and using food in tourism. However, they are often conditioned by bureaucratic technicalities that hinder the initiative and action of numerous subjects (54).

The need to ask the third research question arises from the acknowledgment that local food quality is an important factor that often depends on numerous other factors such as government and economic bodies. It is evident that the research aimed at comprising all the defined factors as potential guidelines in recognizing the need for this form of assistance in tourism regions. Therefore, the third research question is as follows (Q3): Which gastronomic identity factors of the studied areas are dependent on the need for financial incentives and aid allocated for food tourism development?

## 3 Methodology

### 3.1 Creating a questionnaire

For the purpose of this research, a questionnaire was created that consisted of three parts. The first part collected data on the socio-demographic characteristics of the respondents with detailed information on education and places of work among other elements (sex, age, residence, level of education, field of education, place of work, and job position). The second part determined the factors relevant to defining the gastronomic identity in order to develop food tourism. For the purpose of the research, the GastroIdentity scale was created based on the research conducted by Suna and Alvarez (8, 9), who dealt with the gastronomic brand in well-developed tourism destinations. First, their scale was used to conduct a pilot study in order to see if the scale fits, and then it was modified into the GastroIdentity scale used here. The scale consists of 25 statements to which the participants responded by indicating their answer on the 5-point Likert scale (from 1- completely disagree to 5- completely agree). In addition, the third part investigated respondents' attitudes toward the need for incentives for the development of hospitality and tourism activities, in the form of yes/no questions.

### 3.2 Research implementation

The research was conducted in two tourism regions—one in the Republic of Serbia (hereinafter RS) and the other in Bosnia and Herzegovina (hereinafter BIH):

- In RS, the research was conducted in the northern region of the Autonomous Province of Vojvodina including the gravitational area of the city of Novi Sad with Fruška Gora Mountain, as an important tourist area that attracts tourists because of its natural beauty, cultural heritage, and spa facilities.
- In BIH, the research was conducted in the area of the central region, which includes the gravitational area of the city of Sarajevo with Jahorina Mountain, as an important tourist area that attracts tourists because of its natural beauty and cultural and sports facilities.

**Figure 1:**
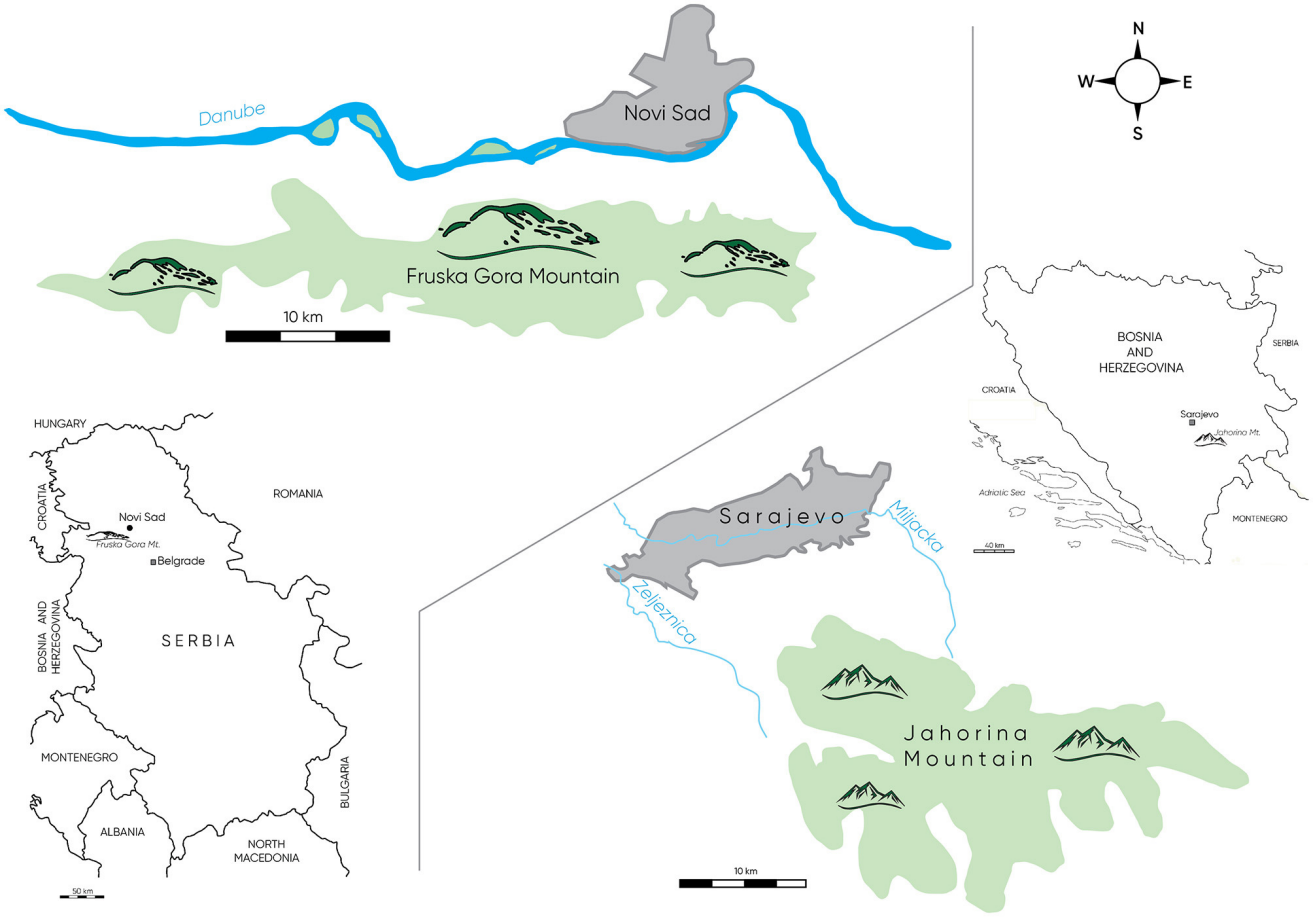
Location of the studied tourism regions (author: Ðerčan, 2023).

In total, 400 questionnaires were distributed in each region, out of which 305 questionnaires from the RS area were collected and processed and 301 questionnaires were from the BIH area. Figure 1 shows the place of work of the respondents included in the study. Most of the questionnaires were administered in the period from 15 June to 15 August 2023. Most of them were distributed in the study in Bosnia and Herzegovina, after which the entries and systematization for processing were carried out. In Serbia, most of the survey questionnaires were distributed via e-mail due to the available database of catering facilities and associates from the fields of hospitality, hotel management, tourism, as well as educational institutions. All respondents were previously informed about the type of research, and they voluntarily agreed to participate.

### 3.3 Statistical data analysis

The data collected through the questionnaire were systematized and processed using R version 4.1.2 software. The first part of the data on the socio-demographic characteristics of the respondents was processed using descriptive statistics. The second part of the questionnaire refers to the analysis of respondents' views on the factors influencing the development of the hospitality and tourism industry in their areas. Exploratory factor analysis (EFA) was applied here. After the EFA, in order to determine the variables that influence the respondents' opinions on whether they need incentives for the development of hospitality and tourism in their region, the method of binary logistic regression was applied.

## 4 Study results

### 4.1 Analysis of results from the Republic of Serbia

In order to comprehensively analyze the obtained data, the starting point is the analysis of the socio-demographic characteristics of the respondents, the results of which are shown in Table 1. Based on the results of the descriptive statistical analysis, in the sample of 305 respondents, there were 54.8% male and 45.2% female respondents. When it comes to the age structure of the respondents, a vast majority of participants were in the group up to 30 years of age (46.6%), followed by the age group 41 + (28.9%) and 31 to 40 (24.6%).

**Table 1:** Socio-demographic characteristics of the respondents in the Republic of Serbia (*n* = 305).

**Variables**	**Categories**	**n**	**Percentage**
Sex	Male	167	54.8
	Female	138	45.2
Age	Up to 30 years old	142	46.6
	31–40 years old	75	24.6
	41+ years old	88	28.9
Residence	Urban	198	64.9
	Rural	107	35.1
Level of education	Secondary education	147	48.2
	College degree	33	10.8
	Bachelor's degree	81	26.6
	Master's/PhD	44	14.4
Field of education	Economy, law, management	50	16.4
	Technology, agriculture, chemistry	27	8.9
	Tourism, hospitality	153	50.2
	Other	75	24.6
Place of work	Travel agency, tourism organization	45	14.8
	Accommodation facility	82	26.9
	Catering facility	137	44.9
	Educational institution in the field of tourism and catering	41	13.4
Job position	Operational job	183	60.0
	Education	49	16.1
	Operational management	35	11.5
	Senior management	38	12.5

Regarding the place of residence, more than half of them (64.9%) live in urban areas. According to the level of education, almost half of the respondents completed secondary education (48.2%), whereas only 10.8% obtained a college degree. Slightly more than half (50.2%) have an education in the field of hospitality and tourism. It can be seen that 44.9% of the respondents work in catering facilities (restaurants, pubs, bars etc.), 26.9% work in accommodation facilities (hotels, motels, and apartments), 14.8% of the respondents are employed in travel agencies and tourism organizations, while 13.4% work in educational institutions in the field of tourism and hospitality. Based on the results, it can be observed that among the respondents, the majority (60%) perform operational jobs (receptionist, tour guide, hostess, chef, waiter, sommelier, bartender, etc.).

In order to get to the key factors that influence the respondents' views on whether they need incentives for the development of the hospitality and tourism industry in their region, a descriptive statistical analysis was first performed (Table 2).

**Table 2:** Descriptive statistics of variables used in factor analysis in the Republic of Serbia (*n* = 305).

**Variables**	**Mean**	**Std. deviation**
The area has authentic gastronomic culture and tradition	4.151	0.8173
The area has attractive gastronomic culture and tradition contributing to the development of the hospitality and tourism sector	3.980	0.8809
The area is characterized by dishes and beverages with unique and recognizable local flavors	4.246	0.8203
The area has rich gastronomic heritage cherished by the local population	4.030	0.8712
Food culture is a reflection of the lifestyle of the local population	4.134	0.8726
Homemade and authentic dishes are prepared from local ingredients	3.928	0.8856
Gastronomic culture and tradition of the place reflect local culture	4.092	0.8340
Gastronomic culture and tradition are a reflection of all the nations living here	4.157	0.8782
Local agricultural and gastronomic products are nutritionally superior to mass-produced ones	4.184	0.8515
Restaurateurs ensure food quality by applying hygiene and health safety standards and systems	3.679	0.8555
Local food has the food quality label that guarantees quality	3.342	0.9924
Local gastronomic and agricultural food products have a constant sensory quality	3.423	0.9875
Local agricultural and food products are of the same nutritional quality throughout the year	3.391	1.0056
The offer of the dishes in the restaurants is local and authentic	3.554	0.9689
In their menus, restaurateurs specifically indicate authentic and local gastronomic products	2.928	1.1009
The offer of local food is complemented by the kindness of the staff toward customers	3.633	0.9120
There are agricultural farms that sell their products directly to tourists	3.826	0.9897
Pronounced seasonality in the agricultural farm business negatively affects the quality of business in the hospitality and tourism sector	3.656	0.9543
There are gastronomic events where local products are presented	4.249	0.7969
During their stay at the destination, tourists have the opportunity for participating in the preparation of local dishes	3.272	1.1390
Gastronomic events contribute to the branding of local gastronomy	4.170	0.8529
Presenting local agricultural food and gastronomic products at events contributes to the establishment of the concept of sustainable tourism	4.023	0.8446
There are food routes visited by tourists	2.931	1.1320
When visiting gastronomic events, tourists get to know the gastronomic culture and traditions of the micro-region where they are staying	3.895	0.8519
Restaurateurs positively perceive and support gastronomic activities that take place outside the catering establishment	3.603	1.0653

Based on the results of the descriptive statistics of the variables used in the factor analysis, it can be seen that the respondents mostly agree with the statement that gastronomic events organized in their region present local products (Mean = 4.249) as well as with the statement that the area is characterized by dishes and beverages that have unique and recognizable local tastes (Mean = 4.246) and that locally produced gastronomic products have better nutritive quality than the mass-produced ones (mean = 4.184). The lowest level of agreement is expressed regarding the statements that there are food routes visited by tourists (Mean = 2.931), that tourists enjoy the opportunity for participating in the preparation of local dishes during their stay (Mean = 3,272), and that the local and authentic products have been specifically branded in the menus (Mean = 2.928). Harman's single-factor test was used to determine whether the results obtained were biased (55). The results of this test show that by assessing all the variables using principal component analysis, the total extracted variance was below 50% (32.1%), i.e., that there were no significant bias effects. First, the justification of the application of factor analysis was tested using the Kaiser–Meyer–Olkin test and Bartlett's sphericity test (Table 3).

**Table 3:** The Kaiser-Meyer-Olkin (KMO) and Bartlett's test of justification of factor analysis—Republic of Serbia.

**Kaiser-Meyer-Olkin measure of sampling adequacy**		**0.912**
Bartlett's test of sphericity	Approx. chi-square	3,675.809
	df	300
	Sig.	0.000

The obtained value of the Kaiser–Meyer–Olkin coefficient is 0.912, which far exceeds the recommended value of 0.6 (56), and it can be considered adequate to use factor analysis for a given set of variables. These results were confirmed by Bartlett's test of sphericity (χ^2^ = 3,675.809; df = 300; *p* < 0.05), based on which it can be concluded that there is a statistically significant correlation between the observed variables. In the correlation matrix, it was observed that there was a sufficient number of coefficients whose values were >0.3 as well as a sufficient number of statistically significant correlation coefficients.

In order to accurately identify the factors found in the correlation matrix, the principal components (PCA) method was used. This method identifies groups of variables that have, on the one hand, high correlation within the group, and on the other hand, low correlation values with other groups. The extracted factors were then rotated using Varimax rotation and the values for five extracted factors were shown (Table 4).

**Table 4:** Total variance explained—Republic of Serbia.

**Components**	**Initial Eigenvalues**			**Extraction sums of squared loadings**			**Rotation sums of squared loadings**		
	**Total**	**% of variance**	**Cumulative %**	**Total**	**% of variance**	**Cumulative %**	**Total**	**% of variance**	**Cumulative %**
1	8.680	34.720	34.720	8.680	34.720	34.720	5.037	20.146	20.146
2	2.490	9.959	44.679	2.490	9.959	44.679	3.076	12.305	32.451
3	2.089	8.354	53.033	2.089	8.354	53.033	2.958	11.833	44.284
4	1.246	4.985	58.018	1.246	4.985	58.018	2.340	9.362	53.645
5	1.183	4.733	62.751	1.183	4.733	62.751	2.276	9.106	62.751

Based on the values shown in the previous table, it can be seen that five factors with an eigenvalue >1 were singled out using the principal components method. These five singled-out factors explain 62.75% of the total variation. Cronbach alpha coefficient obtained from all the factors ranges from 0.738 to 0.908, which is greater than the recommended level of 0.6 (56). In the following segment of the analysis, the factor loading after rotation was observed (Table 5). In order to label adequate factors, factor saturation was observed for each statement in order to determine its role and importance. In presenting the results of the factor analysis, a statement was considered to be significant for a certain factor if the primary saturation was >0.40 (57).

**Table 5:** Factor loading after rotation in the Republic of Serbia.

**Variables**	**Component**				
	**1**	**2**	**3**	**4**	**5**
**Gastronomic culture and heritage**					
The area is characterized by dishes and beverages with unique and recognizable local flavors	0.779				
The area has rich gastronomic heritage cherished by the local population	0.776				
The area has an authentic gastronomic culture and tradition	0.775				
Gastronomic culture and tradition reflect all the nations living here	0.759				
Food culture is a reflection of the lifestyle of the local population	0.724				
The area has an attractive gastronomic culture and tradition contributing to the development of the hospitality and tourism sector	0.708				
The gastronomic culture and tradition reflect local culture	0.701				
Homemade and authentic dishes are prepared from local ingredients	0.673				
**Food quality**					
Local agricultural and food products are of the same nutritional quality throughout the year		0.797			
Local gastronomic and agricultural food products have a constant sensory quality		0.780			
Local regional food has the food quality label that guarantees quality		0.758			
Restaurateurs maintain food quality by applying hygiene and health safety standards and systems		0.692			
Local agricultural and gastronomic products are nutritionally superior to mass-produced ones		0.599			
**Gastronomic events**					
There are gastronomic events where local products are presented			0.740		
Gastronomic events contribute to the branding of local gastronomy			0.732		
Presenting local agriculture, food and gastronomic products at events contributes to the establishment of the concept of sustainable tourism			0.718		
When visiting gastronomic events, tourists get to know the gastronomic culture and traditions of the micro-region where they are staying			0.600		
Tourism culinary activities					
There are food routes that tourists visit				0.768	
During their stay at the destination, tourists have the opportunity for participating in the preparation of local dishes				0.669	
Restaurateurs positively perceive and support gastronomic activities that take place outside the catering establishment				0.661	
**Local food vendors**					
Pronounced seasonality of the agricultural farm business negatively affects the quality of business in the hospitality and tourism sector					0.741
The offer of dishes in the restaurants is local and authentic					0.665
The offer of local food is complemented by the kindness of the staff toward customers					0.660
In their menus, restaurateurs specifically indicate authentic and local gastronomic products					0.540
There are agricultural farms selling their products directly to tourists					0.514
Cronbach's alpha	0.908	0.836	0.815	0.744	0.738

Based on the results shown in Table 5, it can be seen that the first factor has the highest values of factor loadings for eight statements, the analysis of which labels the factor as gastronomic culture and tradition. The second factor is defined through five statements related to various aspects of food quality, and this factor can be labeled as food quality. The third factor loading is determined by four statements related to the importance of events in the observed region, and this factor is labeled as gastronomic events. The fourth factor loading is mostly determined by three statements related to the tourist aspect of the region's gastronomic offer; accordingly, the fourth factor is defined as tourist culinary activities. The last factor is defined through five statements related to the offer of local food in the region, and this factor is defined as local food vendors. In Table 6, it can also be seen that the factor loadings of the statements have different values for different factors, and based on their values, those that have the greatest influence on each factor can be singled out. The highest factor loading of gastronomic culture and tradition has the statement that implies that the area is characterized by dishes and beverages that have unique and recognizable local tastes (0.779). The second factor is mostly determined by the statement that local agricultural and food products are of the same nutritional quality throughout the year (0.797) and the least by the statement that local agricultural and gastronomic products are nutritionally better than mass-produced products (0.599). The gastronomic events factor is mostly saturated by the statement that there are local events where local products are presented (0.740), while the tourist culinary activities factor has the highest load on the statement that implies that there are food tourism routes visited by tourists (0.768). The fifth factor is mostly saturated by the statement that pronounced seasonality in the business of agricultural farms negatively affects the quality of business in the hospitality and tourism sector (0.741).

**Table 6:** Logit model results.

**Factors**	**B**	**S.E**.	**Wald**	**df**	**Sig**.	**Exp (B)**	**95% C.I. for Exp (B)**	
							**Lower**	**Upper**
Gastronomic culture and heritage	−0.559	0.322	3.011	1	0.083	0.572	0.304	1.075
Food quality	1.190	0.427	7.784	1	0.005	3.288	1.425	7.589
Gastronomic events	−0.559	0.329	2.883	1	0.089	0.572	0.300	1.090
Tourism culinary activities	0.723	0.445	2.641	1	0.104	2.061	0.862	4.931
Local food vendors	−0.086	0.334	0.066	1	0.797	0.918	0.477	1.766
Constant	−4.534	0.653	48.149	1	0.000	0.011		

In the following part of the analysis, the impact of the singled-out factors on the attitude of the respondents was examined, namely, whether they think that incentives are needed for the development of the hospitality and tourism industry in the region (1-yes and 2-no). For this purpose, the method of binary logistic regression was applied. Based on the results of the omnibus test, it can be concluded that the coefficients show that the model is well adjusted to the data [χ^2^(17) = 15.915, *p* = 0.007]. This result was also confirmed by the results of the Hosmer Lemeshow test [χ^2^(8) = 6.324, *p* = 0.611]. The results of the binary logistic regression are shown in Table 6.

Based on the results of the binary logistic regression shown in Table 6, it can be observed that food quality stands out as a statistically significant factor influencing the attitude of the respondents regarding the need for incentives for the development of hospitality and tourism industry in the region at the significance threshold of 1% (*p* < 0.01). At the significance threshold of 10% (*p* < 0.10), gastronomic culture and tradition and gastronomic events stand out as statistically significant factors influencing respondents' attitudes regarding the need for incentives for the development of the hospitality and tourism industry in the region.

### 4.2 Analysis of results from the territory of Bosnia and Herzegovina

Next, the data collected from the questionnaires distributed in Bosnia and Herzegovina were analyzed. First, the socio-demographic characteristics of the respondents were analyzed (Table 7). The descriptive statistical analysis results determined that in the survey of 301 respondents, the representation of male respondents was 57.8% and the representation of female respondents was 42.2%. When it comes to the age structure of respondents, the largest percentage of respondents is in the group up to 30 years old (42.9%), followed by the participation of the group from 31 to 40 years old and older (31.9%) and then group aged 41 years and above (25.2%).

**Table 7:** Socio-demographic characteristics of respondents in Bosnia and Herzegovina (*n* = 301).

**Variables**	**Categories**	***N***	**Percentage**
Sex	Male	174	57.8
	Female	127	42.2
Age	Up to 30 years old	129	42.9
	31–40 years old	96	31.9
	41+ years old	76	25.2
Residence	Urban	250	83.1
	Rural	51	16.9
Level of education	Secondary education	141	46.8
	College degree	45	15.0
	Bachelor's degree	89	29.6
	Master's/PhD	26	8.6
Field of education	Economy, law, management	77	25.6
	Technology, agriculture, chemistry	19	6.3
	Tourism, hospitality	75	24.9
	Other	130	43.2
Place of work	Travel agency, tourism organization	40	13.3
	Accommodation facility	86	28.6
	Catering facility	162	53.8
	Educational institution in the field of tourism and catering	13	4.3
Job position	Operational job	197	65.4
	Education	16	5.3
	Operational management	51	16.9
	Senior management	37	12.3

According to the place of residence, the majority of respondents (83.1%) live in urban areas. According to the level of education, most of the respondents completed secondary education (46.8%), while only 8.6% completed Master's/PhD studies. One quarter (24.9%) have an education degree in the field of tourism and hospitality, one quarter (25.6%) in the field of economics, and 43.2% are from other fields. According to the respondents' place of work, it can be seen that slightly more than half (53.8%) work in catering facilities (restaurants, pubs, bars etc.), while only 4.3% work in educational institutions in the field of tourism and hospitality. Based on the results, it can be seen most of the respondents perform operational jobs (receptionist, tour guide, hostess, chef, waiter, sommelier, bartender, etc.), i.e., 65.4% (Table 7).

In order to get to the key factors that influence the views of respondents on whether they need incentives for the development of hospitality and tourism activities in their region, as well as in the case of the Republic of Serbia, respondents were asked 25 questions. Answers to all questions are defined on the 5-point Likert scale (1- completely disagree to 5- completely agree). The results of the descriptive statistical analysis of the mentioned questions are presented in Table 8.

**Table 8:** Descriptive statistics of the variables used in the factor analysis in Bosnia and Herzegovina.

**Variables**	**Mean**	**Std. deviation**
The area has authentic gastronomic culture and tradition	3.731	1.0539
The area has an attractive gastronomic culture and tradition that contributes to the development of the hospitality and tourism sector	3.754	0.9930
The area is characterized by dishes and beverages that have unique and recognizable local flavors	3.738	1.0524
The area has a rich gastronomic heritage that the local population cherishes	3.635	1.0921
Food culture is a reflection of the lifestyle of the local population	3.771	1.0379
Homemade and authentic dishes are prepared from local ingredients	3.631	1.0955
Gastronomic culture and tradition of the place reflect local culture	3.824	1.0028
Gastronomic culture and tradition are a reflection of all the nations living here	3.801	1.0646
Local agricultural and gastronomic products are nutritionally superior to mass-produced products	3.841	1.0682
Caterers ensure food quality by applying hygiene and health safety standards and systems	3.621	0.9501
Local food has food quality label that guarantees quality	3.116	1.1357
Local gastronomic and agricultural food products have a constant sensory quality	3.256	1.0055
Local agricultural and food products are of the same nutritional quality throughout the year	3.282	1.0909
The offer of dishes in the restaurants is local and authentic	3.246	1.1428
In their menus, restaurateurs specifically indicate authentic and local gastronomic products	3.296	1.1898
The offer of local food is complemented by the kindness of the staff toward customers	3.734	1.0782
There are agricultural farms that sell their products directly to tourists	3.073	1.1894
Pronounced seasonality in the business of agricultural holdings negatively affects the quality of business in the hospitality and tourism sector	3.136	1.0415
There are gastronomic events where local products are presented	3.060	1.1789
During their stay at the destination, tourists have the opportunity for participating in the preparation of local dishes	2.671	1.2281
Gastronomic events contribute to the branding of local gastronomy	3.465	1.1268
Presenting local agricultural food and gastronomic products at events contributes to the establishment of the concept of sustainable tourism	3.625	1.0273
There are food routes visited by tourists	2.850	1.1723
When visiting gastronomic events, tourists get to know the gastronomic culture and traditions of the micro-region where they are staying	3.495	1.1094
Restaurateurs positively perceive and support gastronomic activities that take place outside the catering establishment	3.312	1.1025

Based on the results of the descriptive statistics of the variables used in the factor analysis, it can be seen that the respondents mostly agree with the statement that local agricultural and gastronomic products are nutritionally richer than mass-produced ones (Mean = 3.841) as well as with the statement that gastronomic culture and tradition of an area reflect local cultural aspects (Mean = 3.824) and that all nations that inhabit the area of interest are part of the gastronomic culture and tradition (Mean = 3.801). The lowest agreement level is expressed with the statements that there are food tourism routes visited by tourists (Mean = 2.931), that tourists enjoy the opportunity for participating in local dish preparation during their stay (Mean = 3,272), as well as that agricultural farm products are sold directly to tourists (Mean = 3.073). As with the previous questionnaire, Harman's single-factor test was used to determine whether the obtained results were biased (55). The results of this test show that by testing all variables using principal component analysis, the total extracted variance was below 50% (39.436%), i.e., that there were no significant bias effects. The justification for applying factor analysis in this case was also tested using the Kaiser–Meyer–Olkin test and Bartlett's test of sphericity (Table 9).

**Table 9:** Kaiser–Meyer–Olkin (KMO) and Bartlett's test of justification of factor analysis in Bosnia and Herzegovina.

**Kaiser–Meyer–Olkin measure of sampling adequacy**		**0.936**
Bartlett's test of sphericity	Approx. chi-square	4,108.883
	df	300
	Sig.	0.000

The obtained value of the Kaiser–Meyer–Olkin coefficient is 0.936, which far exceeds the recommended value of 0.6 (56), and it can be considered adequate to use factor analysis for a given set of variables. These results were confirmed by Bartlett's test of sphericity (χ^2^ = 4,108.883; df = 300; *p* < 0.05), based on which it can be concluded that there is a statistically significant correlation between the observed variables. The correlation matrix also showed that there was a sufficient number of coefficients whose values are >0.3, as well as a sufficient number of statistically significant correlation coefficients. In order to precisely identify the factors found in the correlation matrix, as in the previous case, the method of principal components (PCA) was used. The extracted factors were then rotated using Varimax rotation, and the values for the four extracted factors are shown (Table 10).

**Table 10:** Total variance explained in Bosnia and Herzegovina.

**Components**	**Initial Eigenvalues**			**Extraction sums of squared loadings**			**Rotation sums of squared loadings**		
	**Total**	**% of variance**	**Cumulative %**	**Total**	**% of variance**	**Cumulative %**	**Total**	**% of variance**	**Cumulative %**
1	10.447	41.787	41.787	10.447	41.787	41.787	5.358	21.432	21.432
2	1.941	7.762	49.550	1.941	7.762	49.550	3.717	14.870	36.302
3	1.479	5.915	55.464	1.479	5.915	55.464	3.123	12.492	48.794
4	1.242	4.968	60.432	1.242	4.968	60.432	2.910	11.638	60.432

Based on the values shown in the previous table, it can be seen that four factors that had an eigenvalue >1 were singled out using the principal components method. These four singled-out factors explain 60.432% of the total variation. Cronbach alpha coefficient obtained from all the factors range from 0.813 to 0.921, which is greater than the recommended level of 0.6 (56). The following table shows the factor loadings after rotation (Table 11).

**Table 11:** Factor loading after rotation in Bosnia and Herzegovina.

**Variables**	**Component**			
	**1**	**2**	**3**	**4**
**Gastronomic culture and heritage**				
The area has authentic gastronomic culture and tradition	0.767			
The gastronomic culture and tradition of the place reflect local culture	0.764			
The area has an attractive gastronomic culture and tradition that contributes to the development of the hospitality and tourism sector	0.762			
Gastronomic culture and tradition are a reflection of all the people who live here	0.760			
Homemade and authentic dishes are prepared of local ingredients	0.745			
The area is characterized by dishes and beverages that have unique and recognizable local flavors	0.723			
The area has a rich gastronomic heritage cherished by the local population	0.691			
Food culture is a reflection of the lifestyle of the local population	0.670			
**Culinary activities**				
During their stay at the destination, tourists have the opportunity for participating in the preparation of local dishes		0.800		
There are food routes visited by tourists		0.693		
There are gastronomic events where local products are presented		0.679		
There are agricultural farms that sell their products directly to tourists		0.619		
Restaurateurs positively perceive and support gastronomic activities that take place outside the catering facility		0.513		
**Gastronomic events and local food vendors**				
Gastronomic events contribute to the branding of local gastronomy			0.779	
Presenting local agricultural food and gastronomic products at events contributes to the establishment of the concept of sustainable tourism			0.756	
When visiting gastronomic events, tourists get to know the gastronomic culture and traditions of the micro-region where they are staying			0.657	
Local agricultural and gastronomic products are nutritionally superior to mass-produced ones			0.535	
The offer of local food is complemented by the kindness of the staff toward customers			0.464	
The offer of dishes in the restaurants is local and authentic			0.425	
In their menus, restaurateurs specifically indicate authentic and local gastronomic products			0.403	
**Food quality**				
Restaurateurs establish food quality by applying hygiene and health safety standards and systems				0.764
Local food has the food quality label that guarantees quality				0.683
Local agricultural and food products are of the same nutritional quality throughout the year				0.619
Local gastronomic and agricultural food products have a constant sensory quality				0.592
Cronbach's alpha	0.921	0.829	0.813	0.830

Based on the results shown in Table 11, it can be seen that the first factor has the highest values of factor loadings for eight statements, the analysis of which can define the name of the factor gastronomic culture and tradition. The second factor is defined through five statements related to various activities organized in the region, and this factor is defined as culinary activities. The third factor loading is determined by seven statements related to the importance of events in the observed region and the offer of local products, and this factor is defined as gastronomic events and local food vendors. The fourth factor loading is saturated by four statements related to different aspects of food quality; accordingly, the fourth factor is defined as food quality.

Table 11 also shows that the highest factor loading of gastronomic culture and tradition belongs to the statement that the area has an authentic gastronomic culture and tradition (0.767). The second is mostly saturated by the statement that tourists during their stay at the destination have the opportunity to participate in the preparation of local dishes (0.800) and the least by the statement that restaurateurs positively perceive and support gastronomic activities held outside the catering facility (0.513). The factor gastronomic events and local food vendor is mostly saturated by the statement that refers to the fact that gastronomic events contribute to the branding of local gastronomy (0.779), while the factor food quality has the highest load on the statement that implies that restaurateurs establish food quality by applying hygiene and health and safety standards and system (0.768). In the following part of the analysis, the impact of the selected factors on the attitude of the respondents was examined and whether according to them incentives are needed for the development of hospitality and tourism activities in the region (1-yes and 2-no). For this purpose, the method of binary logistic regression was applied. Based on the results of the Omnibus test, it can be concluded that the coefficients show that the model is well adjusted to the data [χ^2^(17) = 10.109, *p* = 0.039]. This result was also confirmed by the results of the Hosmer Lemeshow test [χ^2^(8) = 3.198, *p* = 0.921]. The results of the binary logistic regression are shown in Table 12.

**Table 12:** Logit model results in Bosnia and Herzegovina.

**Factors**	**B**	**S.E**.	**Wald**	**df**	**Sig**.	**Exp (B)**	**95% C.I.for EXP (B)**	
							**Lower**	**Upper**
Gastronomic culture and heritage	−0.111	0.163	0.469	1	0.493	0.895	0.650	1.230
Culinary activities	−0.429	0.173	6.135	1	0.013	0.651	0.464	0.914
Gastronomic events and local food vendors	−0.252	0.166	2.317	1	0.128	0.777	0.561	1.075
Food quality	−0.185	0.159	1.353	1	0.245	0.831	0.608	1.135
Constant	−1.951	0.183	113.482	1	0.000	0.142		

Based on the results of the binary logistic regression shown in Table 12, it can be observed that as a statistically significant factor influencing the attitude of respondents on whether the incentives are needed for the development of hospitality and tourism in the region, at the significance threshold of 5% (*p* < 0.01), there is only the culinary activity factor. Other observed factors, in this case, were not statistically significant.

## 5 Discussion

The identification of the gastronomic identity factors that influence the development of food tourism in the areas of RS and BIH was greatly helped by obtaining the viewpoints of the relevant respondents who participated in the survey. A closer look into the structure of the respondents from both areas shows that the majority of the employees are under the age of 30 years, followed by an overall significant share of respondents older than 30 years, among whom almost half only completed secondary education. Considering the pre-set research priorities, it is important to emphasize that in both tourist locations, there is a greater number of employees in urban areas compared to rural areas. The two samples differ in the field of education since more than half of the respondents from Serbia completed their education in the field of hospitality and tourism, which is extremely important for this type of research, while in Bosnia and Herzegovina, this share was almost twice as low, with the majority respondents working in the hospitality industry whose education is not directly related to hospitality and tourism. A significant share of respondents from both tourist locations are employed in catering establishments (restaurants, pubs, bars etc.) and the least in educational institutions in the field of tourism and hospitality, which also affects the structure of respondents' answers. According to the previously mentioned level of education, which is leading, it is important to note the job positions taken by the respondents which, in both cases, mostly include operational jobs (receptionist, tour guide, hostess, chef, waiter, sommelier, bartender, etc.), with a share of over fifty percent.

The analysis of the obtained data related to the set of data relevant to the first research question (Q_1_) shows that employees from the territory of RS emphasize that there are local gastronomic events presenting local products as well as that the area is characterized by dishes and beverages which have unique and recognizable local tastes. Respondents express the lowest level of agreement regarding the food routes visited by tourists, which has been expected because they are not defined in the RS in relation to the wine routes (58).

Based on the results obtained from Bosnia and Herzegovina, it can be observed that the respondents share the opinion that among all the mentioned characteristics, local agricultural and gastronomic products are nutritionally better than products of mass production. This clearly confirms their awareness of the quality of local food in relation to other products, which has also been corroborated by other studies (59, 60). They also point out that part of the local gastronomic culture and tradition, accessible and visible to tourists, is a true reflection of the culture of the entire region and the local area. These results confirm the potential for the development of food tourism, which is certainly one of the prerequisites for the development of tourism in general but also of the particular tourist area (61). The lowest level of agreement was noted, as was the case with the respondents from the Republic of Serbia, regarding the established food routes visited by tourists, which both tourist regions should work on (62) since they represent a complete arrangement that unites all types of producers and providers of gastronomic experience of a certain area, with a very attractive opportunity for tourists to participate in the preparation of gastronomic specialties and in active learning about the gastronomic culture of a nation (21).

A further analysis gave an affirmative answer to the second research question (Q_2_), namely, the created scale is adequate for this type of research and it helped define certain important factors for both regions. The analysis of the data for the RS singled out and defined five factors: gastronomic culture and tradition, food quality, gastronomic events, tourist culinary activities, and local food vendors. The analysis of the data obtained in Bosnia and Herzegovina singled out and defined four factors, namely, gastronomic culture and tradition, culinary activities, gastronomic events, local food vendors, and food quality. By looking at the existing gastronomic identity and the obtained factors for both tourist locations, it was agreed that gastronomic culture and tradition are indispensable elements contributing to the improvement of food tourism, and they are acknowledged as critical factors in other research as well (8, 9).

The survey also answered the third research question (Q_3_), which was related to incentives. The analysis shows that the significant factor, according to the respondents, when considering the need for incentives for the development of catering and tourism activities, including food tourism in the RS region, is conditioned by the respondents' perception of local gastronomic culture and traditions and gastronomic events. In contrast to the results from the RS area, in Bosnia and Herzegovina, they are conditioned by the respondents' views on culinary activities, which requires raising awareness regarding the possibility of improvement and better placement of the gastronomic heritage and the use of all available economic and cultural potentials in the tourism of Bosnia and Herzegovina.

## 6 Conclusion

The study of the factors of gastronomic identity that influence the development of food tourism, observed in the examples of tourist destinations in Southeast Europe and from the point of view of employees in catering and tourism, as well as educational institutions that educate future creators and actors of the catering and tourism offer, it has been concluded that there are certain differences in the two observed tourist areas. Within the RS, as an important element of the gastronomic identity for the development of tourism, gastronomic events have been singled out as important presenters of local gastronomy, with unique and recognizable local tastes. Employees from the BIH tourist area emphasized the nutritional quality of their products, as a significant potential in tourism in relation to mass-produced ones, as well as the gastronomic culture and tradition, which is very different from the other observed tourist market. The food routes proved to be the least recognized, which is due to their non-existence. AElements can be seen as a downside of the overall tourist product, with a special emphasis on food tourism development. The research showed that the set GastroIdentity scale was appropriate for this type of study, highlighting gastronomic culture and tradition as the most important factor in the development of food tourism within both areas, assigning positive connotation to the authenticity and uniqueness of the food offer. This factor stands out as important in recognizing the need for incentives for the development of hospitality and tourism activities, and at the same time, food tourism in the RS region, together with the gastronomic events of the region. While in the areas of Bosnia and Herzegovina, it is more conditioned by culinary activities, which still are to be adequately implemented in the region.

In the developing areas, tourism is expected to be a part of an economic transformation, which will have a positive influence on the development of the gastronomic identity of the micro-region (63). Therefore, there are significantly important theoretical and practical implications in terms of set problems and results. Hence, these implications will continue to be part of the vivid and detailed discussion.

### 6.1 Theoretical contribution of the research

Rapid improvements in development policies that include local gastronomy led to gastronomic identity becoming the focus of various studies in recent years (64). In previous studies, analyzed tourist areas were primarily set in the developed tourism micro-areas (8, 9, 65). In realizing and implementing gastronomic identity in tourism, the studies were centered around tourists and less on the actors. The focus of this research is the popularization and need to highlight local gastronomy as one of the main resources of a tourist destination, which has been corroborated by many scientific studies with the same aim (61, 66–68). In developing countries, there have not been a lot of studies dealing with the restaurateurs' opinions, gastronomic identity factors, and food tourism. Therefore, this research summarizes significant theoretical principles that will provide theoretical background for the future treatment, investigation, and interpretation of this particular topic (8).

### 6.2 Managerial contributions of the research

There is no available research on gastronomic identity in terms of factors that impact the potential representation for the development of food tourism in Novi Sad and Sarajevo. Therefore, this research provides an insight into the perception of hospitality and tourism offer, including the elements that employees in catering and tourism, as well as education, see as important for the development of food tourism, which can help in their better placement and use. The obtained data also provides an insight into the weaknesses of gastronomic potentials, which can be compensated for by following the example of many developed gastronomic tourism destinations, such as the creation of food routes. Based on the obtained results, clear strategies can be defined for action on the tourist market and better placement of the available resources of the gastronomic identity. The results show that there is a greater appreciation of the variables that are associated with specific contribution of catering facilities in the creation of gastronomic identity than the general claims that are associated with gastronomic culture and tradition. It can be concluded that the gastronomic identity is identified, but to a large extent, it is not popularized and implemented in the business of the hospitality industry. Hence, this significantly interrupts the constitution of the sustainable development concept (8).

In designing this research, the authors had the intention to make the results applicable in practice. The GastroIdentity scale can be used for the identification of set goals effects during and after the improvement plans in Novi Sad, Sarajevo, and other tourist destinations in the Republic of Serbia and Bosnia and Herzegovina, which would significantly contribute to food tourism development in both regions and further.

This research aimed to promote further studies dealing with the identification of gastronomic identity factors. These studies should incorporate tourists, decision-makers, top management of hospitality and tourism companies, and managers of educational programs. Observing the subjects' perceptions, a distinct multidisciplinary model will be obtained in order to improve the approach to the perception of the factors of gastronomic identity that impact the development of food tourism (9). All these claims serve as an excellent basis for setting up long-term gastronomy and tourism offer strategies.

### 6.3 Suggestions for further research

In subsequent research, emphasis could be placed on certain groups of actors in tourism, important for the operation and placement of appropriate tourist and gastronomic products. In this way, one could see potential shortcomings within the system, that is, the operation and placement of food that is part of the cultural identity of the tourist market. In the same way, the study of the factors included in this research could be carried out among tourists as important participants in the recognition of gastronomic identity through various tourist activities, as well as the way of consumption, as an important element in achieving the ultimate goals and profit from the food sales.

## Data availability statement

The original contributions presented in the study are included in the article/supplementary material, further inquiries can be directed to the corresponding author.

## Ethics statement

Ethical approval was not required for the studies involving humans because all respondents were previously informed about the type of research and voluntarily participated in it. The studies were conducted in accordance with the local legislation and institutional requirements. Written informed consent for participation was not required from the participants or the participants' legal guardians/next of kin in accordance with the national legislation and institutional requirements because All respondents were previously informed about the type of research and voluntarily participated in it.

## Author contributions

BKP: Conceptualization, Validation, Writing—original draft, Writing—review & editing. MP: Conceptualization, Writing—original draft. NŠ: Conceptualization, Writing—original draft. SŠ: Conceptualization, Data curation, Writing—review & editing. PM: Formal analysis, Writing—original draft. VI: Data curation, Writing—review & editing. JM: Data curation, Investigation, Writing—review & editing. SP: Data curation, Investigation, Writing—review & editing. DTek: Methodology, Writing—review & editing. BÐ*: Data curation, Software, Writing—review & editing. DTeš: Conceptualization, Writing—review & editing. SGJ: Data curation, Formal analysis, Writing—original draft.
